# Insights into the Biology and Therapeutic Applications of Neural Stem Cells

**DOI:** 10.1155/2016/9745315

**Published:** 2016-03-16

**Authors:** Lachlan Harris, Oressia Zalucki, Michael Piper, Julian Ik-Tsen Heng

**Affiliations:** ^1^The School of Biomedical Sciences, The University of Queensland, Brisbane, QLD 4072, Australia; ^2^Queensland Brain Institute, The University of Queensland, Brisbane, QLD 4072, Australia; ^3^The Harry Perkins Institute of Medical Research, Perth, WA 6009, Australia; ^4^The Centre for Medical Research, Perth, WA 6009, Australia

## Abstract

The cerebral cortex is essential for our higher cognitive functions and emotional reasoning. Arguably, this brain structure is the distinguishing feature of our species, and yet our remarkable cognitive capacity has seemingly come at a cost to the regenerative capacity of the human brain. Indeed, the capacity for regeneration and neurogenesis of the brains of vertebrates has declined over the course of evolution, from fish to rodents to primates. Nevertheless, recent evidence supporting the existence of neural stem cells (NSCs) in the adult human brain raises new questions about the biological significance of adult neurogenesis in relation to ageing and the possibility that such endogenous sources of NSCs might provide therapeutic options for the treatment of brain injury and disease. Here, we highlight recent insights and perspectives on NSCs within both the developing and adult cerebral cortex. Our review of NSCs during development focuses upon the diversity and therapeutic potential of these cells for use in cellular transplantation and in the modeling of neurodevelopmental disorders. Finally, we describe the cellular and molecular characteristics of NSCs within the adult brain and strategies to harness the therapeutic potential of these cell populations in the treatment of brain injury and disease.

## 1. NSCs during Development of the Cerebral Cortex

The development of the mammalian cerebral cortex follows stepwise production of neurons, then glial cells, including astrocytes and oligodendrocytes from local NSCs. Early during embryonic development, cells of the central nervous system are derived from the neuroectoderm, which is organised as a neural tube. Over time, the neural tube invaginates to form structures including the prosencephalon, from which emerge the telencephalon and diencephalon. The cerebral cortex arises from the dorsal telencephalon (also known as the pallium), while the ventral telencephalon (also known as the subpallium) gives rise to the basal ganglia (reviewed in [1]). NSCs from the dorsal and ventral telencephalon are critical to the generation of the two main classes of cerebral cortex neurons, the excitatory projection neurons which signal using glutamate as their neurotransmitter and the inhibitory interneurons that use *γ*-amino butyric acid (GABA). Excitatory projection neurons are born from local NSCs residing within the dorsal telencephalon, and these neurons migrate radially to position themselves appropriately within the developing cortex and hippocampus (reviewed in [2]). Different subtypes of excitatory cortical projection neurons are generated in a temporal sequence, so as to generate defined layers I to VI. On the other hand, inhibitory interneurons are born from NSCs residing within the ventral telencephalon. These neurons undergo long-distance, tangential migration to populate the dorsal cortical structures.

A remarkable feature during cerebral corticogenesis is the synchronous development and complementary positioning of temporally derived interneurons from the ventral telencephalon and projection neurons from the dorsal telencephalon, such that functional neural circuits are established between excitatory projection neurons and their appropriate inhibitory interneuron counterparts [2–4]. In all, the characteristic six-layered structure of the neocortex features neurons in layers V and VI which project to subcortical targets (subcerebral projection neurons and corticothalamic projection neurons, resp.), neurons in layers II and III which largely project to other cortical areas (corticocortical projection neurons, as well as callosal neurons which project to the contralateral hemisphere and whose axons comprise the corpus callosum), and neurons in layers I and IV that largely form axonal connections within the cortical hemisphere. Within each layer, cortical interneurons adopt a variety of unique dendritic morphologies so as to modulate projection neuron firing. Detailed accounts of the formation of cortical projection neuron and interneuron subtypes are the subject of several excellent reviews [2–5].

*The Diversity of NSCs within the Embryonic Cerebral Cortex*. NSCs are defined by their capacity to self-renew, as well as be able to generate neurons, astrocytes, and oligodendrocytes. In contrast, not all neural progenitor cells (NPCs) exhibit self-renewal capacity. The diversity of NSCs and NPCs (i.e., cells which generate neurons but are not necessarily self-renewing) is, in part, responsible for the diversity and relative population densities of neuronal subtypes within the cerebral cortex. Early in the formation of the cerebral cortex, the dorsal telencephalon comprises a uniform layer of neuroepithelial cells. The local NSCs of the germinal ventricular zone (VZ), which lines the vesicular lumen, initially undergo self-renewing, proliferative divisions. At approximately mid-gestation in rodents, a subset of NSCs transition to become lineage-restricted NPCs and accumulate as a secondary proliferative layer above the VZ, described as the subventricular zone (SVZ). A population of NSCs undergo neurogenic divisions to form an early, transient neuronal layer above the SVZ, known as the preplate (PP). As corticogenesis progresses, newborn neurons split the PP layer to form an outer marginal zone (MZ), an underlying cortical plate (CP), and a subplate (SP). The MZ comprises a small (1–3%) population of distinct progenitors [6] as well as Cajal-Retzius cells which derive from the cortical hem/antihem and septum [7] and which secrete Reelin, an essential factor for cortical layering (reviewed in [8]). Over time, newborn cortical neurons continue to migrate into the CP, resulting in its progressive enlargement. Notably, cortical neurons are added to each of the CP layers in a temporally specified manner, such that neurons which occupy deep layers IV and V are generated early during corticogenesis, while neurons of superficial layers (IV, III, and II) are generated later. Eventually, the cell-sparse MZ forms layer I, while the VZ/SVZ compartment is progressively depleted and reduced to a single-cell layer of ependymal cells, with the exception of the lateral wall of the cortical SVZ which continues to support a niche of resident glial-like NSCs which generates neurons into adulthood (discussed in Section 2).

**Figure 1:**
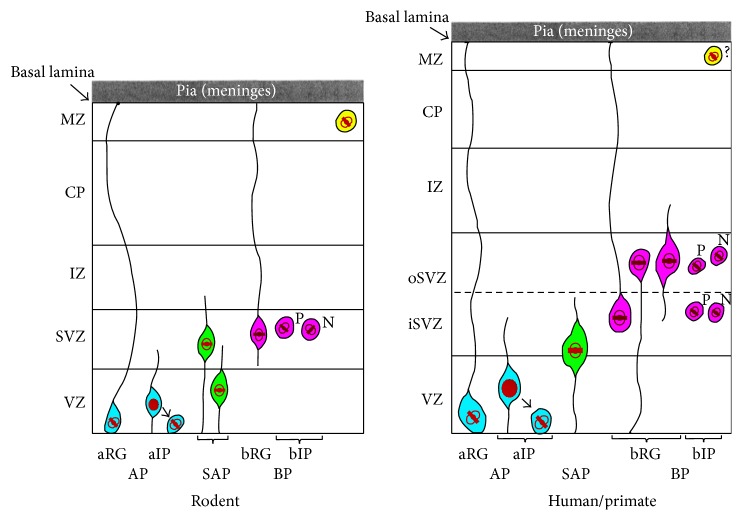
Summary of progenitor subtype diversity within the rodent and human/primate brain. Apical Progenitor (AP) cells (light blue) include apical Radial Glia (aRG) which attach to the basal lamina and apical Intermediate Progenitor (aIP) cells which have short processes. Both types of APs are defined by their mitotic division at the apical surface. Subapical Progenitor (SAP) cells (coloured green) are defined by their ventricular contact and abventricular mode of cell division. Basal Progenitor (BP) cells (magenta) are defined by their basal mitoses and comprise basal Radial Glia (bRG) cells attached to the basal lamina as well as basal Intermediate Progenitor (bIP) cells which undergo a proliferative division (labelled “P”) or neurogenic divisions (labelled “N”), as indicated. A yellow coloured marginal zone progenitor is represented in rodent cortex. In the human/primate cortex, AP and SAP cell types have been identified, while three types of bRGs have been identified including those with a basal attachment, an apical attachment, or only emanating short processes. The bIPs cell types which undergo proliferative or neurogenic divisions have been described in the iSVZ and oSVZ. The presence of MZ progenitor cells within the human/primate cortex remains to be clarified. VZ: ventricular zone, SVZ: subventricular zone, IZ: Intermediate Zone, CP: cortical plate, MZ: marginal zone, iSVZ: inner subventricular zone, and oSVZ: outer subventricular zone as presented. Relative sizes of rodent and human/primate compartments are not drawn to scale. See text for further details.

In the last two decades, significant progress has been made to describe the cellular heterogeneity of embryonic NPCs and NSCs in the dorsal telencephalon (Figure 1). Notably, three main types of progenitor cells have been identified on the basis of their relationship with the apical surface of the dorsal telencephalon (located immediately adjacent to the ventricular lumen) relative to the superficial basal lamina of the MZ, as well as their distinct cellular/molecular features: (i) Apical Progenitors (APs); (ii) Basal Progenitors (BPs); and, more recently, (iii) Subapical Progenitors (SAPs) [47–49].

APs are NSCs that remain in contact with the luminal wall and form adherens junctions with other Apical Progenitors. APs are also identified by the apical location of their mitoses, are able to translocate their nuclei along the vertical axis in a cell cycle dependent fashion (termed interkinetic nuclear migration (INM) [50, 51]), and exhibit apicobasal polarity [49, 52]. Interestingly, APs show a temporal relationship whereby the earliest APs identified within the telencephalon are the neuroepithelial (NE) cells that undergo proliferative, symmetric divisions to expand the local pool of progenitors. As corticogenesis progresses, NE cells adopt an asymmetric mode of cell division generating apical Radial Glial (aRG) cells as well as apical Intermediate Progenitor (aIP) cells (first described as short neural precursors [53]) or, more infrequently, neurons. NE cells and aRG cells are capable of proliferative divisions, while aIPs undergo a single round of symmetric, neurogenic division to generate two identical daughter neurons [53].

The BPs, the second type of progenitor cell within the cortex, are identifiable by their detachment from adherens junction complexes within the VZ, their location within the SVZ, their expression of the transcription factor TBR2 (also known as EOMES), and their capacity to undergo basal rather than apical mitotic divisions [54, 55]. BPs are the products of cell division by NE cells and aRG cells and comprise two main cell types, namely, basal Intermediate Progenitors (bIPs) and basal Radial Glia (bRG). BPs can undergo symmetric, neurogenic divisions that ultimately deplete the pool of SVZ progenitors. However, the capacity for proliferative divisions by BPs and the size of the relative population of BP subtypes are significantly different in lissencephalic (smooth) brained rodents, compared to the gyrencephalic (convoluted) brains of primates such as humans [47]. The expansion of BPs in the human cortex that can undergo proliferative divisions has been suggested to constitute an important cellular basis for human cortical expansion and gyrification [56]. Indeed, recent findings demonstrate the preponderance of bRG cells in the human ventral forebrain, which generate large numbers of cortical interneurons [57].

More recently, a new type of cortical progenitor, SAPs, was recognised to be distinct from APs and BPs, owing to the abventricular location of their mitoses and their ventricular contact [48]. These SAPs are capable of proliferative divisions and are more abundant in the ventral versus the dorsal telencephalon. Furthermore, SAPs appear to be more numerous in the cortices of gyrencephalic brains of ferrets and sheep, compared with the lissencephalic cortex of the marmoset. Given their recent discovery by Pilz and colleagues [48], the precise contribution by SAPs to the cellular diversity of cortical neurons remains to be clarified. However, their abundance in both the dorsal and the ventral telencephalon suggests that SAPs are likely to play a significant role in cortical neuron development.

While excitatory cortical projection neurons are generated from NSCs of the dorsal telencephalon, inhibitory cortical interneurons are largely generated from the germinal zones of the ventral telencephalon. In mice, the ventral telencephalon is organised into several prominent structures termed ganglionic eminences, each with distinct NSC and NPC populations that generate specific neuronal subtypes. For example, NPCs of the lateral ganglionic eminences (LGE) generate striatal projection neurons and interneurons destined for the olfactory bulb, while NPCs residing within the medial ganglionic eminences (MGE) generate cortical interneurons that invade the dorsal telencephalon, as well as local projection neurons of the globus pallidus. Also, the anterior entopeduncular area (AEP) is recognised as a source of interneurons that populate the dorsal cortex via tangential migration [58]. Notably, it was within the considerably large SVZ of the mouse ventral telencephalon that Pilz and colleagues discovered SAPs undergo mitoses at basal positions, away from the ventricular surface of the telencephalon [48].

While it is clear that the primary source of cortical interneurons in rodents appears to be the ventral telencephalon, the developmental origin of human cortical interneurons appears to involve both the ventral and dorsal cortex, with evidence supportive of a limited contribution by dorsal cortical progenitors. Letinic and colleagues presented the first evidence that prominent numbers of interneuron progenitors could be found within the dorsal cortex, as identified by their expression of Dlx1, Dlx2, and Mash1 [59]. This finding was supported by subsequent studies demonstrating the propensity for dorsally derived cortical cells to differentiate into subtypes of interneuron [60–64]. However, Hansen and coworkers [57, 65] more recently reported that DLX2-expressing cells of the dorsal cortex are not colabelled with Ki67, a marker of cell proliferation, or incorporate the DNA-synthesis marker BrdU in studies with brain slice cultures. A similar conclusion was drawn by Ma and colleagues in their studies of human and primate (macaque) cortex, since they found that cultured slices of monkey dorsal cortex yielded an extremely low proportion of GABAergic neurons that arise from at least one cell division (marked by BrdU incorporation) [65]. These new findings thus provide compelling evidence to support the notion that the vast majority of human cortical interneurons are of a subcortical origin and that interneuron progenitors in the cortex are postmitotic. Interestingly, in cases of human holoprosencephaly (HPE) with severe ventral forebrain hypoplasia, it was reported that only subpopulations of cortical interneurons (namely, those which express either NOS1, NPY, or SST) were absent, while calretinin-positive interneurons were still detected [66]. In such cases it would appear that certain interneuron subtypes could arise from the dorsal cortex, at least in situations in which the ventral forebrain is severely compromised. The capacity for cortical interneuron production in the human brain in the context of development and disease remains to be clarified.

*Insights into the Molecular Regulation of NSCs within the Embryonic Cerebral Cortex*. Over the course of embryonic cortical development, the timing of NSC proliferation and neurogenesis is guided by cell extrinsic and cell intrinsic factors. The cerebrospinal fluid (CSF) courses through the ventricular system of the neural tube to deliver numerous signalling factors that influence the proliferative potential of cortical NSCs [67]. As early as embryonic day (E) E8.5–E9.5 in the mouse, Sonic Hedgehog (Shh), Fibroblast Growth Factor (Fgf), and Bone Morphogenetic Proteins (Bmps) establish gradients across the rostrocaudal, lateromedial, and dorsoventral telencephalon [2]. Such extrinsic signals are interpreted by embryonic cortical cells to induce NSC expression of genes encoding transcription factors such as* Lim-homeodomain 2* (*Lhx2*),* Forkhead Box G1* (*FoxG1*),* Paired Box Domain 6* (*Pax6*), and* Empty Spiracles Homologue-1* and* Empty Spiracles Homologue*-*2* (*Emx1* and* Emx2*) in a region-specific manner. Notably, Lhx2 is detected in the entire telencephalon except for the dorsal midline, while Foxg1, Pax6, and Emx1 are expressed in cells of the dorsal telencephalon, and Emx2 is expressed throughout the telencephalon [68].

The expression patterns for these abovementioned transcription factors reflect their instructive roles for NSC proliferation and neurogenesis. For example, Lhx2 specifies cortical and hippocampal cell fates, and studies of knockout mice reveal that its absence results in the expansion of adjacent structures, including the midline structures known as the cortical hem and antihem [69, 70]. More recent investigations of* Lhx2* deficiency using lineage-specific cre-driver mice have revealed its role in NSC proliferation and neurogenesis. In studies of conditional (loxp) mice crossed with* Nestin*-*cre* to delete* Lhx2* throughout the developing nervous system, Lhx2 was found to regulate progenitor proliferation and neurogenesis through *β*-catenin signalling [71]. Deletion of* Lhx2* in telencephalic progenitors using* Emx1-cre* mice led to the formation of olfactory cortex rather than lateral cortex in a critical developmental window (E10.5) in embryonic mouse development [72]. The activity of Lhx2 appears to involve the transcriptional regulation of downstream target genes, such as* Pax6*, as revealed by Shetty and coworkers, who demonstrated that loss of* Lhx2* in mouse embryos from E11.5 onwards led to the loss of distinct neurocircuitry (namely, the barrel cortex) which accompanied changes in the regional identity of the cortex and which appeared to phenocopy* Pax6* deficiency [73].

In the case of Foxg1, its expression within the E9.5 embryo is observed as a high-rostrolateral-to-low-caudomedial gradient. Deletion of* Foxg1* in the mouse results in repatterning of the cortical field to cortical hem and hippocampus, together with the concomitant loss of cortical plate neurons [74]. In newborn cortical neurons, the precise timing of* Foxg1* expression is critical for their migration from the IZ to the CP through a mechanism which, in part, involves modulation of* Unc5D* expression [75]. In addition, the sequential production of Cajal-Retzius cells, deep layer neurons followed by upper layer neurons requires* Foxg1*, with its selective loss resulting in the commensurate disruption of this temporal sequence for the production of cortical glutamatergic neurons [76, 77]. Hence, Lhx2 and Foxg1 are necessary to specify the identity of cortical NSCs.

In contrast to Lhx2 and Foxg1, studies of mouse corticogenesis reveal that Pax6 is critical for dorsal* versus* ventral telencephalic identity [78–81].* In situ* hybridisation studies reveal a regionalised pattern for Pax6 in the dorsal telencephalon, with high rostrolateral expression and low caudomedial expression. In contrast, Emx1 and Emx2 expression is detected in an opposing (low rostrolateral expression and high caudomedial expression) gradient [82]. Pax6 was recognised to be critical for establishing cortical identity, due to studies of the mouse mutant* small eye* that revealed that* Pax6*-deficiency led to ectopic expression of ventral telencephalic genes by dorsal telencephalic NSCs, including* Mash1*,* Gsh2*, and* Dlx1/2* [78–81]. Mutations to* Emx1/2* result in a reduction in the size of the cortex [83], but studies of compound* Pax6/Emx2* double-mutant embryos reveal that both these genes are required in concert for establishing the identity of dorsal cortical NSCs, since their compound loss results in the lack of the dorsal telencephalon, and an expansion of ventral telencephalic domains across the entire cortex [84]. Together, these studies provide examples of cell intrinsic factors that specify the identity of NSCs during cortical development.

In addition to the functions for Pax6 in cortical regionalisation, additional studies have underscored its importance in regulating the transition between APs and BPs within the embryonic cortex, as well as its role in driving neurogenesis. For example, Pax6 activates expression of the proneural basic helix-loop-helix (bHLH) transcription factor Neurog2 in APs to instruct their neuroprogenitor fate and to drive neuronal subtype specification in postmitotic neurons [79]. Pax6 also orchestrates the proliferation of APs and promotes their asymmetric division to expand the pool of BPs within the embryonic cortex [85–87]. Homozygous mutant mice for* Pax6* display a selective loss of cortical neurons destined for upper layers [88–90].

The role and interplay of intrinsic factors in cortical neuron specification and subtype identity is perhaps best exemplified in studies of Neurog2 and its related family members Neurog1 and Ascl1. Both Neurog2 and Neurog1 are specifically expressed by APs and, to a lesser extent, BPs [91]. Furthermore, studies with lineage tracer (*Neurog2* ^*EGFPKI*^) mice indicate that* Neurog2* is expressed in early postmitotic neurons which have exited S/G2/M-phases of the cell cycle [91]. On the other hand, Ascl1 is detected predominantly in NSCs of the ventral telencephalon. Loss of Neurog2 leads to the reduction of early-born glutamatergic neurons destined for layers V and VI of the mouse cortex, and this phenotype is exacerbated in* Neurog1/Neurog2* double-mutant mice [79, 92]. In contrast, in* Ascl1* loss-of-function mutants there is depletion of NSCs of the ventral telencephalon and medial ganglionic eminence, as well as their neuronal progeny [93]. Interestingly, loss of* Neurog2*, or both* Neurog1* and* 2*, leads to ectopic expression of Ascl1 by dorsal telencephalic progenitors, as well as the subsequence ectopic expression of ventral telencephalic genes, including* Dlx1*,* Dlx2*,* Dlx5*, and the GABAergic neurotransmitter genes* Gad1* and* Gad2* [92]. In support of the notion that Neurog2 suppresses Ascl1 in the cortex, these ventral telencephalic markers are not detected in* Neurog2*/*Ascl1* double-mutant embryos [79]. Thus, the specification of glutamatergic projection neurons versus GABAergic interneurons by NSCs is governed by the activities and interplay of transcription factors, such as the proneural bHLH proteins (please refer to these articles [2, 3, 58, 94] for further details).

Studies of proneural bHLH transcription factors in the embryonic cortex can also provide insights into the timing of neurogenesis versus gliogenesis by NSCs. For example, loss of both* Neurog2* and* Ascl1* leads to a significant reduction in neuronal production coupled with premature initiation of gliogenesis within the embryonic cortex [95]. The expression of Neurog2 or Ascl1 in NSCs is required to maintain their neurogenic potential and prevent activation of gliogenesis [95]. A proposed mechanism for this dual role was reported by Sun and colleagues who found that Neurog1 induced neurogenesis through the direct activation of neuronal genes, while suppressing glial differentiation through sequestration of the transcriptional coactivating factor CREB-binding protein (CBP). This sequestration prevented the association of CBP with Smad1 and Stat transcription factors and thereby the activation of the promoters of astrocyte-specific genes including* S100β* and* glial fibrillary acidic protein* (*Gfap*) [96]. More recently, studies of the zinc finger transcriptional repressor Rp58 (also known as Znf238) have identified its role in cortical development. Notably, Rp58 is detected in NSCs and postmitotic neurons of the dorsal telencephalon, and homozygous loss of* Rp58* leads to a disruption of NSCs and BPs [97], premature gliogenesis [98], and defective neurogenesis [97, 99, 100] within the embryonic cortex. Interestingly, Rp58 can bind* Neurog2*-like gene regulatory sequences, repressing candidate gene expression as well as antagonising the functions of* Neurog2*-type transcriptional activators [99]. Furthermore, Rp58 directly suppresses the expression of* Neurog2* [101] as well as* Neurog2*-target genes, such as* Rnd2* [99]. Thus, these findings suggest that Rp58 regulate the development of newborn postmitotic neuronal progeny in part through mechanisms involving neurogenins, but the precise mechanisms that underlie the function of Rp58 in APs and BPs remain to be clarified.

Insights into the regulation of neurogenesis versus gliogenesis by NSCs can also be gleaned from studies of the* nuclear factor one* (NFI) family of transcriptional regulators (comprising* Nfia*,* Nfib*,* Nfic*, and* Nfix*) [102–104]. These proteins bind target DNA sequences so as to activate or repress candidate gene transcription in a context-specific manner (reviewed in [105]). Nfia, Nfib, and Nfix are detected within the VZ of the dorsal telencephalon [106, 107], and studies of homozygous mutant mice revealed that the loss of* Nfia*,* Nfib*, or* Nfix* led to an expansion of NSCs (marked by Pax6 expression), but without an accompanying increase in BPs (marked by Tbr2) [108–110]. Curiously,* Nfi* deficiency resulted in a reduction in gliogenesis, marked by Gfap expression [109, 111, 112]. To account for these interesting phenotypes, molecular studies have begun to clarify the activities of Nfis in the coordination of NSC expansion and neuronal-glial differentiation. For example, a study by Namihira and colleagues reported that Notch signalling-induced Nfia expression in cortical NSCs was coincident with derepression of the* Gfap* regulatory region (marked by dissociation of the DNA methyltransferase Dnmt1 from this locus), which was coincident with promoter occupancy by the activator Stat3 [113]. While these observations suggest that NFIs regulate gliogenesis through indirect mechanisms, their roles in NSC expansion could be linked to their transcriptional regulatory activities on target genes that govern stem cell identity. This is supported by recent findings that demonstrate that the expression of stem cell-associated genes* Ezh2* and* Sox9* is directly suppressed by Nfib [114] and Nfix [109]* in vitro* and that* Ezh2* and* Sox9* expression is elevated in* Nfib* and* Nfix* knockout mouse embryos. Interestingly, the expression of the two Notch-induced stem cell regulators, namely, the* Hairy-Enhancer-of-Split* (*Hes*) genes* Hes1* and* Hes5*, is also elevated in* Nfia* and* Nfib* knockout mice, further suggesting a role for both NFIs in suppressing NSC self-renewal [110]. Collectively, these lines of evidence suggest that NFIs orchestrate the timely progression of NSC proliferation, neurogenesis, and gliogenesis during cortical development, likely by directly suppressing NSC genes and modulating* Gfap* expression through an indirect mechanism. Taken together, these examples provide insight into the underlying mechanisms which regulate embryonic NSCs. Given the relatively recent discovery of the extensive cellular heterogeneity of NSCs in the developing cerebral cortex, as well as the potential differences between rodent and human NSCs [115] (Figure 1), the molecular mechanisms underlying the functions for each distinct AP and BP subtype remain a significant topic of interest.

## 2. NSCs in the Adult Cerebral Cortex

A subset of embryonic NSCs persists into the postnatal and adult mammalian brain throughout life. Much like in the developing brain, the biology of these adult NSC populations has primarily been studied using rodent models. The biology of adult NSCs in rodents is, at face value, very similar to embryonic NSCs, with the major point of difference being that adult NSCs are long-lived and largely quiescent. In rodents, the two adult neurogenic niches are the subgranular zone (SGZ) of the hippocampal dentate gyrus and the SVZ lining the lateral ventricles. In the SGZ, NSCs give rise to Intermediate Progenitors and then immature granule neurons, which integrate into existing hippocampal circuitry (reviewed by [116]). In the adult SVZ, NSCs (B1 cells) are located in the walls of the lateral ventricles neighbouring the hippocampus, cortex, striatum, and septum [117]. These cells give rise to Intermediate Progenitor cells, then immature neurons that migrate towards the olfactory bulb where they generate different types of interneurons (reviewed by [118]).

### 2.1. The Developmental Origin of Adult NSCs

The dogma concerning the developmental origin of SGZ and SVZ adult NSCs has been that NSCs within the SGZ arise from the dentate neuroepithelium during embryonic development and that NSCs within the SVZ represent a continuation of embryonic progenitors from the lateral wall that become specified during the early postnatal period. Recently however, both of these views have been challenged.

The view that SGZ NSCs solely originate from the dentate neuroepithelium was promoted by a pioneer study of rat dentate gyrus development from Altman and Bayer [119]. In this study, it was shown that the structural development of the dentate gyrus or primary neurogenesis [120] began when precursor cells migrated away from the dentate neuroepithelium to establish an abventricular site of proliferation from which the dentate gyrus would later form. Some of these cells, they argued, persisted to form the SGZ during the second postnatal week, thereby implicating the dentate neuroepithelium as the source of SGZ precursors. In 2013, Li and colleagues [121] directly challenged this idea using genetic manipulations and fate mapping experiments. Based on previous observations that Shh signalling is necessary for SGZ formation, but not for the formation of granule cell layer of the dentate gyrus [121, 122], they generated reporter mice in which cells receiving Shh signalling would be labelled. Curiously, labelled cells were mostly present in the ventricular zone of the ventral hippocampus from E14. Time course experiments using a cre-dependent reporter demonstrated that these cells in the ventral hippocampus migrated to the dentate gyrus through a septotemporal route. Reporter cells were found to label a subset of proliferating cells in the SGZ shortly after birth and also in 12-month-old mice. These results demonstrated that at least some SGZ NSCs arise from the ventral hippocampus and are specified during early development. However, as not all SGZ stem cells were labelled in the reporter mice in this study, this suggests that SGZ stem cells likely have multiple developmental origins. An interesting line of inquiry will be to establish whether there is a relationship between the developmental origin of SGZ stem cells and the emerging functional heterogeneity of this population [123].

Two recent studies have also challenged the origin of Type B1 stem cells in the rodent SVZ. Because prior studies had shown that B1 cells are derived from NSCs from multiple regions of the germinal ventricular zone surrounding the lateral ventricles during development [124, 125] and exhibit similar morphology and gene expression patterns, this had suggested a linear lineage relationship from NE cells to aRG cells to B1 cells. While this hypothesis would predict that B1 cells are specified during the early postnatal period when aRG cells become depleted, both Fuentealba and colleagues [126] and Furutachi and colleagues [31] used label-retention assays, such as thymidine analog injections, to demonstrate that the majority of B1 cells became quiescent (retained the analog label) if injected at E14, but not after this point. Further experiments using a retroviral barcoding paradigm confirmed these observations, as only aRG cells that had been transfected with the retroviral library prior to E14-E15 shared a clonal relationship with B1 cells in the postnatal brain. By delimiting the spatial and temporal origin of B1 cells, these studies have enabled a platform for future investigations to interrogate the signalling pathways involved in specification. As a window to these possibilities, in Furutachi and colleague's [31] study they identified p57 as a key molecule in generating quiescent NSCs. High expression of p57 during embryogenesis predicted a quiescent state for these cells in the adult brain, and loss of p57 led to reduced numbers of quiescent NSCs. This finding suggests that forced expression of p57 could be used to manipulate NSC number in the adult brain.

Together, these studies have shed a new light on the developmental origin of NSCs within the adult cerebral cortex. The most surprising and unifying element of these studies is that the NSCs within these niches are specified early during development, during mid-neurogenesis within the fetal brain. These findings emphasise the tight temporal regulation of adult NSC specification and suggest that the specification of these cells may not be solely due to stochastic processes during the early postnatal period. Indeed, the broad significance of these findings relates to enhancing our understanding of the developmental origin of adult NSCs in rodents and extending these investigations into primate brain development, which will be key to comprehending the basic biology of these cells in the human brain and so being able to harness their potential for use in regenerative medicine.

### 2.2. Regulation of Adult NSCs

Arguably just as important as studying the developmental origin of adult NSCs is to understand the niche factors and molecular signals that maintain these cellular populations throughout life. Excessive proliferation of adult NSCs (or loss of quiescence) leads to premature depletion and reduced neurogenesis in the long term [11, 127–129]. Conversely, quiescent NSCs must acutely respond to stimuli such as neural activity by proliferating and generating new neurons. The abundance of different niche and molecular factors that control this process of quiescence/proliferation/differentiation is a testament to how finely balanced this process is. Here, we review some of these niche and molecular cues.

#### 2.2.1. The Niche Microenvironment

The spatially restricted nature of neurogenesis in the SGZ and SVZ of the adult brain suggests that there are important local cues that are released to maintain NSC populations. The structural organisation of the niche and the morphology of NSCs support this idea. For example, in the hippocampus, clusters of NSCs are located close to the tips of capillaries [130]. Likewise, B1 cells in the SVZ have a long basolateral process that terminates on blood vessels and a thin apical tip that protrudes into the ventricular space and so is in direct contact with the CSF [131]. Moreover, most SVZ NSCs are located in the highly vascularised lateral side of the lateral ventricles [132]. Some of the factors that are released from vascular/endothelial cells and which regulate neurogenesis include the growth factors Vegf [133] and Pedf [134], the hormone erythropoietin [135], and neurotrophin NT-3 [136].

Interestingly, the cellular source of some of the most important and canonical niche signals that regulate neurogenesis, such as Notch, Wnt, and Shh pathways, is largely unknown. Some of these signals may come from the vasculature or CSF, but they could also come from other cellular components within the niche. For example, coculture of NSCs with niche astrocytes promotes neurogenesis, but this process is not evident when NSCs are cultured with astrocytes from nonneurogenic regions [137], suggesting that niche astrocytes secrete/express some of these important signalling molecules. Local microglia may also secrete ligands in response to exercise to promote neurogenesis [138]; likewise, ablation of neuronal progenitors in the hippocampus (Type 2 cells) or SVZ (Type A cells) through AraC treatment promotes NSC division, demonstrating that these progenitors are also a source of cellular feedback within the niche [117, 139]. In the hippocampus, neural activity also plays an important role in niche homeostasis. GABA released from parvalbumin-expressing interneurons maintains adult NSC in a quiescent state and inhibits self-renewal. As parvalbumin-expressing interneurons are activated by mature granule neurons of the dentate gyrus, this network may therefore suppress neurogenesis during periods of high local activity [20]. As another example, signalling through Nmdar promotes integration of newborn neurons into the existing circuitry [140]. Thus, neurotransmitter signalling within the hippocampus modulates neural activity, providing a local circuitry mechanism that influences the hippocampal NSC niche.

#### 2.2.2. Molecular Regulation of NSC in the Adult Brain

There are many molecular regulators of NSC in the adult brain. An exhaustive discussion of all the molecules and signalling pathways that regulate NSC within the adult brain is beyond the scope of this review. Rather, here we highlight major factors that play a role in this process, which are listed in Table 1. These molecular regulators of adult NSC biology can broadly be defined as extrinsic or intrinsic factors or, alternately, grouped as signalling pathways in cases where the relationships between molecules are understood.

In adult NSCs, similar to their embryonic counterparts, some of the most well-established signalling nodes are the Notch, Bmp, and Wnt pathways. The first of these two pathways, Notch and Bmp, promote NSC quiescence [11, 128, 129], whereas the Wnt pathway promotes symmetric division of adult NSCs [14]. Another large cohort of molecules implicated in adult NSC biology via loss- or gain-of-function experiments are transcription factors. Groups of these proteins, such as members of the bHLH [35], T-box [43], Sox [41], and Nfi transcription factor family members [39], control large suites of genes and therefore act as master regulators over cellular processes such as quiescence, fate commitment, and differentiation.

Of increasing interest to the field is how epigenetic modifications modulate adult NSC behaviour. Chromatin modifications such as DNA methylation of proximal promoters affect the accessibility of chromatin and therefore transcription [141]. For example, the activity of Dnmt3a, a member of the Dnmt family that confers methylation of the 5th position of cytosine (5mC), is crucial for expression of neurogenic genes in adult NSCs [22]. Conversely Tet1, which demethylates cytosine residues by converting 5mC to 5-hydroxymethylcytosine (5hmC), is also crucial for normal adult hippocampal neurogenesis [27]. Indeed, the 5hmC mark is highly enriched in the brain and increases in the hippocampus with age [142]. Together, these examples demonstrate the important balance between methylation and demethylation during adult neurogenesis.

Looking forward, the study of noncoding RNAs will also be an important area in the adult neurogenesis field. Testament to this is a blunt experimental approach taken by Cernilogar and colleagues [143] where they deleted the RNAse III enzyme Dicer in neural tissue. This enzyme is required for the processing and generation of microRNAs and small interfering RNAs that function to silence the expression of specific protein coding transcripts. In this preliminary study, deletion of* Dicer* affected levels of the neuroblast marker doublecortin. Moreover, the RNA interference machinery comprising Dicer/Ago2 was enriched in the chromatin of differentiating versus undifferentiated neural progenitor cells. Similarly, the role of long noncoding RNAs (lncRNAs) has also recently implicated in adult neurogenesis. Of the few lncRNAs studied thus far, both negative regulators of neurogenesis, such as* Six3os* and* Dlx1as* [144], and a positive regulator,* Pnky* [145], have been identified.

Overall, the many regulators of SGZ and SVZ NSCs are testament to the inherent complexity of the cell biology of these cell populations. The future challenge will be to continue to characterise the biological regulators of adult NSCs using existing reductionist approaches and, crucially, to then place these findings in their cellular context through systems biology. In doing so, we will then be well placed to identify signalling pathways and molecules that are therapeutic targets for stem cell-based therapies for degenerative conditions. Increasingly, these efforts will also be accelerated by single-cell sequencing technologies. These technologies provide unprecedented insight into the diversity of cell types in adult neurogenic niches. For example, recent studies employing this technology have revealed subpopulations of NSCs that become activated after ischemic brain injury [146] or exposure to growth factors [147].

## 3. The Therapeutic Potential of NSCs

**Figure 2:**
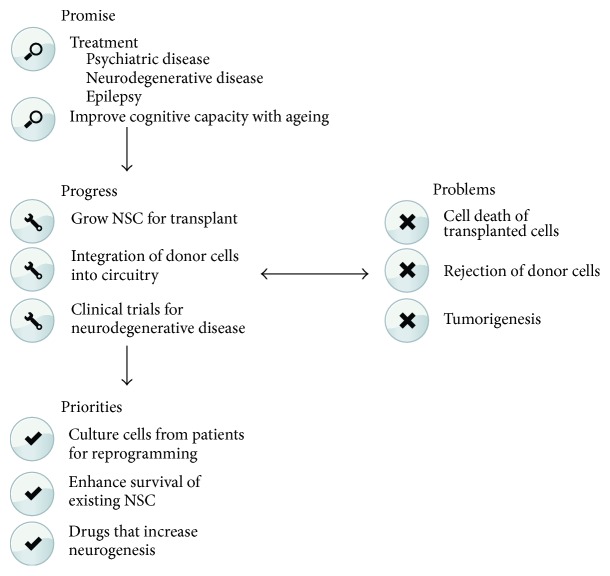
Summary of the promise, priorities, problems, and progress of the therapeutic application of NSCs. This schematic outlines the promise of the therapeutic application of NSCs, several of the priorities for applying NSCs for therapeutic application, some of the problems faced with using NSCs in patients, and finally what progress that has been made in the application of NSCs.

Understanding the molecular signals that regulate neurogenesis during development and within the adult neurogenic niches will help guide the development of NSC based therapies to treat human diseases and conditions. Since monitoring NSCs in human patients is restricted to only correlative postmortem studies, the use of animal models has been essential to gain insight into applying NSCs for therapeutic benefit. For example, an informed view of how fetal NSCs generate interneurons* in vivo* has guided the attempts by some to generate these cells* in vitro* to treat epilepsies through cellular transplantation. Likewise, the dysfunction of adult NSCs is increasingly thought to underlie several major disorders including depression, anxiety, and neurodegenerative disease, although direct causal evidence is lacking. Accelerating endogenous neurogenesis in these contexts may therefore improve patient outcome (Figure 2). Here we discuss some of the most promising applications of NSC based therapies and research.

### 3.1. Insights and Therapeutic Applications Arising from the Study of Fetal Neurogenesis

The study of fetal neurogenesis has led to the evaluation of the potential for these cells and their progeny to treat disease. An elegant study by Baraban and colleagues reported that the transplantation of cells from the E13.5 medial ganglionic eminence (MGE) of mice could reduce the incidence and duration of seizures in a genetic model of epilepsy resulting from a mutation to the potassium channel gene* Kv1.1* [148]. Notably, the authors performed transplantations in presymptomatic (postnatal) mice and observed the widespread distribution and synaptic integration of donor cells that had differentiated into interneurons, suggesting that the presence of donor cells was likely responsible for alleviating seizure-like behaviour when the mice matured to adulthood. While this provides one tantalising experimental therapy for the treatment of epilepsy in humans (deletions to the KV1.1 are associated with one form of human epilepsy), it is unclear if the procedure leads to undesirable behavioural side effects. Nevertheless, these findings reveal the capacity for transplanted fetal cortical cells to disperse broadly within the site of injection so as to modulate excitation-inhibition balance. Such properties of transplanted cells also identify them as potential vectors for the delivery of therapeutic agents.

In addition to fetal cortical sources of cells for transplantation, a study by Gaspard and coworkers pioneered the culture of mouse embryonic stem cells in the presence of a chemical inhibitor of the morphogen Shh to generate cortical glutamatergic neurons in a temporally specified manner [149]. A further study by Espuny-Camacho and colleagues applied analogous cell culture techniques with human embryonic stem (ES) cells and induced pluripotent stem cells (iPSCs) to generate functional cortical pyramidal neurons [150]. By drawing parallels between fetal NSC activities and the progression of cortical neurogenesis, the authors of both studies collectively recognised the potential for their cell-based approaches to model fetal cortical neurogenesis, as well as evaluate the suitability of iPSC and ES cell-derived neurons of distinct subtypes to treat brain injury or disease [151]. To support the viability of this approach, a subsequent study by Michelsen and coworkers demonstrated that mouse ES cell-derived cortical neurons with the molecular properties of visual cortical neurons could restore the axonal connectivity and functional properties of the mouse visual cortex following a lesion [152]. Notably, these ES cell-derived visuocortical-like neurons could not ameliorate the effects of a lesion to the motor cortex, while grafting of motor cortex into the visual cortex lesion also did not lead to restoration of function. These studies demonstrate the importance for matching cell-based sources of neuronal subtypes with the graft site in order to restore region-specific brain function.

Recent technological innovations in cell culture and pluripotent stem cell biology have further converged on the capacity to study the molecular and cellular basis for developmental brain disorders using a three-dimensional culture system [153, 154]. A landmark report by Lancaster and colleagues described an extended rolling culture protocol to generate cerebral organoids from iPSCs [154]. Remarkably, these cerebral organoids recapitulated some of the features of early cortical development, including the spatial organisation of NSCs (marked by PAX6) and BPs (marked by TBR2). Crucially, parallel studies with organoids derived from a patient with microcephaly revealed premature neurodifferentiation, highlighting this as a possible mechanism that may underlie this condition. More recently, Camp and colleagues have applied single-cell gene expression approaches to study both cerebral organoids and fetal neocortical cells in order to identify the similarities and differences between the molecular profiles of cells derived from each of these sources [155]. It was interesting to note in their study that organoids comprised fewer BPs than APs, which could reflect the limitations of organoid culture or discrepancies in the time point between the organoid and fetal cortical tissue. Regardless, this technology is anticipated to accelerate our understanding of the extrinsic and intrinsic factors that influence human cortical development and disease.

While an understanding of fetal neurogenesis can guide our development of cell-based methods to model cortical neurogenesis and developmental brain disorders, a better understanding of the fetal NSC compartment will enable us to identify sources of repair cells which could be mobilized in times of injury or stress. Looking to vertebrates such as zebrafish, a recent excellent study by Barbosa and colleagues described the maintenance of MZ-like stem cells in the periphery of the forebrain from birth to adulthood, and these stem cells can be activated to restore lost brain tissue upon injury [156]. Given the identification of self-renewing MZ progenitors in the embryonic mouse [6] it remains to be determined if common molecular mechanisms could be drawn between MZ NSCs in mice and adult zebrafish NSCs to enable us to engineer mammalian MZ NSCs capable of extensive self-renewal and repair. Such approaches could be extended to account for the molecular mechanisms that underlie the self-renewal capacity of all progenitor cell types in the fetal cortex which have been described in this review.

Knowledge of the molecular mechanisms that drive fetal NSC neurogenesis could also lead to the development of novel cellular substrates for cell transplantation therapy. Guided by insights into the neurogenic programming potential for proneural bHLH factors, Masserdotti and colleagues have recently reported that forced expression of Ascl1 or Neurog2 in postnatal astrocytes and mouse embryonic fibroblasts led to their reprogramming into neurons [157]. However, after prolonged culture astrocytes displayed a loss of neural reprogramming capacity by Neurog2, because of increased competition with the repressor REST complex for the neuronal target gene* NeuroD4* [157]. Such studies are extremely valuable to assess potential sources of patient-derived cells and appropriate culture conditions for neurogenic reprogramming for cell transplantation therapies, as well as the potential to stimulate neurogenesis and repair by endogenous NSCs in the postnatal brain.

### 3.2. Insights and Therapeutic Applications of Studying Adult NSCs

Harnessing existing endogenous populations of adult NSCs could likewise have numerous therapeutic applications in disease and for improving brain function in healthy individuals (Figure 2). For example, the stimulation of neurogenesis in the adult rodent brain is associated with several beneficial effects. Exercise enhances neurogenesis, with positive effects on learning [158, 159]; moreover, the generation of new neurons in the hippocampus is associated with improved spatial memory performance [158, 160–166] and contextual fear learning [167, 168]. Conversely, dysfunction of adult hippocampal NSCs is associated with depression and anxiety and has led to the neurogenic theory of depression [169, 170] that postulates two key features: depression accompanies decreased levels of neurogenesis and, secondly, that restoration of neurogenesis will ameliorate the symptoms. There is widespread evidence to support the first of these claims of this theory. For example, subjecting rodents to repeat restraint stress [171], unpredictable mild stress [172], social defeat stress [173], and social isolation stress [174] results in depression-like behaviours and impaired neurogenesis.

In support of the second claim of the neurogenic theory of depression, electroconvulsive therapy (ECT), which is a well-established tool for treating depression, increases hippocampal neurogenesis in adult rodents [175, 176]. Furthermore, chronic treatment with antidepressant drugs such as fluoxetine, reboxetine, and tranylcypromine also increases hippocampal neurogenesis in adult rodents [177]. In nonhuman primates, chronic fluoxetine and ECT increase hippocampal neurogenesis [178, 179], though whether this correlates with increased hippocampal-dependent learning and memory is not clear. Moreover, rodents with blocked neurogenesis do not recover from depression-like behaviours when antidepressants are chronically administered to them [179–183]. Thus, many treatments for depression enhance adult neurogenesis, and at least in rodents, neurogenesis is required for some aspects of antidepressant function. Confusingly, some recent studies have suggested there is a neurogenesis-independent mechanism of action of antidepressant drugs, showing there is no effect or only modest effects of reducing neurogenesis on the efficacy of antidepressants [180, 183–189]. Thus, while these aforementioned studies highlight an association between neurogenesis and depression, to what extent changes in neurogenesis and depression are causally linked and whether selectively enhancing adult neurogenesis in humans is sufficient or necessary to treat depression are still unclear [190].

Despite these unresolved issues, designing drugs that selectively increase hippocampal neurogenesis stands out as a logical therapeutic strategy in the treatment of depression, particularly since there have been few new antidepressant drugs with novel modes of action in the last decade (Figure 2). Importantly, two decades of research has broadened our understanding of the molecules and signalling pathways that serve to amplify adult hippocampal neurogenesis, providing new therapeutic targets. For example, inhibiting effectors of the Notch and Bmp signalling pathways that mediate NSC quiescence [11, 12, 128, 129] could result in a short-term increase in neurogenesis that may be of therapeutic benefit, though this needs to be considered in parallel with the potential depletion of the quiescent NSC pool that may arise from such a treatment. Other targets include boosting the expression of growth factors that decline with age [191, 192]. Likewise, the discovery that quiescent NSCs uniquely metabolise lipids compared to proliferating NSCs in both the hippocampus [193] and SVZ [194] opens a range of new drug targets. Most promisingly to date, an unbiased screen for compounds that increase neurogenesis in rodents uncovered P7C3 as a potential target [195]. This compound, which promotes the survival of newborn neurons [196], is currently in clinical trials to ameliorate neurodegenerative diseases and it also has known antidepressant effects. With an increased understanding of the molecular pathways regulating adult neurogenesis, drugs that are already on the market could be used off-label if they are known to affect these pathways. An example of this is metformin, a well-tolerated oral medication for diabetes that has recently been shown to have proneurogenic effects in mice [197].

Another potential therapeutic intervention of NSCs is to apply them to ameliorate the age-related cognitive decline of the brain. Ageing is associated with a decline in neurogenesis ([198–201]; plus see review [161]). Ageing seems to affect various aspects of neurogenesis. For example, many studies report a significant age-related decline in cell proliferation [199, 200, 202–205]. The greatest decline in cell proliferation tends to occur by middle-age, and only modest additional declines are reported between middle-age and senescence [201, 206–211]. Not only is cell proliferation affected, but also the capacity of neurons to migrate is compromised with ageing [212, 213].

Are these age-related changes in neurogenesis associated with compromised cognitive capacity? Studies report conflicting results. Aged rats that perform better in the Morris water maze test of spatial learning and memory have more proliferating cells and newborn neurons than age-matched controls [208, 214]. Other studies show no correlation or a negative correlation between proliferating cells and performance [215, 216]. Thus, facilitating neurogenesis during ageing could have beneficial impacts on cognitive function, but further studies are needed.

Several mechanisms have been put forward to explain the age-related decline in neurogenesis. Ageing has been associated with changes in the hippocampal NSC niche vasculature [217]. Moreover, blood-borne factors such as circulating chemokines can inhibit or promote neurogenesis in an age-dependent manner [218]. Growth factors that have important roles in neurogenesis such as Vegf, Fgf2, Bdnf, and Wnt signalling decrease with age [219–221]. Indeed, it is largely these extrinsic factors and alterations to neurogenic niche environment that contribute to the decline in neurogenesis observed with age rather than changes intrinsic to the neural precursors themselves [222]. Recently, chronic administration in aged rats of a peptide known to have neuroprotective properties was shown to restore neurogenesis, synaptic plasticity, and memory [223], suggesting that induction of neurogenesis has beneficial effects on cognition during ageing. Moreover, increasing neuronal activity in the aged brain through seizures can induce quiescent NSCs to reenter the cell cycle and restore proliferation to a level comparable to the one observed in young animals [224]. Thus, targeting key molecules involved in neurogenesis or reactivating NSC offers therapeutic promise in reversing or ameliorating ageing-related changes in brain function (Figure 2).

## 4. Concluding Remarks

In this paper, we have highlighted the cellular and molecular diversity of NSCs in the fetal and adult cerebral cortex. This research is critical as a basis for our understanding of the dynamic properties of embryonic and adult NSCs and how we might be able to manipulate them at the cellular and molecular level. This work has been facilitated by rapid advances in molecular and cellular techniques, as well as sequencing modalities and lineage tracing paradigms. Indeed, this suite of basic research has served as a springboard to drive the therapeutic applications of NSCs towards the treatment of brain injury and disease. While many of these therapeutic approaches are in the early, preclinical stage, it is likely that the knowledge gleaned from the ongoing study of embryonic and adult NSCs will enable the continual refinement of cellular replacement techniques and the identification of therapeutic targets that will lead to real treatments for brain injury and disease in the clinic. Such achievements will realise the promise of NSC research, which has for a long time held the imagination and fuelled the hope for researchers, clinicians, patients, and the broader community.

## Figures and Tables

**Table 1:** Molecular regulators of NSC in adult SGZ and SVZ. Summary of molecular regulators of adult NSC, grouped into ligands, neuropeptides, neurotransmitters, epigenetic, cell cycle regulators, and transcription factors.

Molecule/regulator	Key finding	Ref
Ligands		
Notch	Activation promotes quiescence	[9, 10]
Bmp	Activation promotes quiescence	[11–13]
Wnt	Promotes NSC symmetric division	[14]
Tgf-*β*	Promotes quiescence and survival	[15]

Neuropeptides		
Npy	Induces proliferation, migration, and differentiation of NSC	[16–19]

Neurotransmitters		
GABA	Maintains adult NSC quiescence	[20]

Epigenetic		
Chd7	Maintains adult NSC quiescence	[21]
Dnmt1/3a	Increased expression in differentiating NSC; upregulation favours neurogenic fate	[22, 23]
Gadd45	Required for expression of extrinsic factors from mature granule neurons that modulate neurogenesis	[24]
Hdac2	Required for NSC differentiation and appropriate expression of progenitor markers	[25]
Mbd	Loss-of-function reduces neurogenesis	[26]
Tet1	Positively regulates NSC proliferation	[27]

Cell cycle regulators		
p21	Maintains quiescence and negatively regulates SOX2 expression	[28, 29]
p27	Maintains quiescence	[30]
p57	Maintains quiescence	[31, 32]

Transcription factors		
Foxo3	Maintenance of progenitor cells and quiescence	[33, 34]
Ascl1	Controls neuron fate commitment; overexpression produces oligodendrocytes	[35–38] [39]
Nfix	Maintains NSC quiescence *in vitro*	[39]
Pax6	Maintenance of NSCs	[40]
Sox2	Maintains NSC self-renewal through Shh signalling	[41, 42]
Tbr2	Required for generation of Intermediate Progenitors in DG	[43]
Tlx	Required for NSC self-renewal through WNT and neuron fate commitment through Mash1	[36, 44, 45]
Rest	Maintenance of NSC	[46]
